# Supplementary material for: A location discrete choice model of crime: Police elasticity and optimal deployment
Source: PLoS One. 2024 Mar 12;19(3):e0294020. doi: 10.1371/journal.pone.0294020 (PMC10931527; doi:10.1371/journal.pone.0294020)
Supplement: S1 Appendix — (PDF) [file pone.0294020.s001.pdf]

Appendix S1: Police Time Reassignment Strategies

Reassignment 3

The third reassignment strategy considered tries to reallocate police patrol time from the quadrants that are both the less responsive and report less crimes toward the quadrants that are more responsive and report more crimes. In particularly, from the time that is taken away from the former, we increase by $x\%$ the time that was originally received by the latter. In this case, we try different values of $x \in \{10, 20, 30, 40, 50, 60, 70, 80, 90, 100\}$ for each type of crimes. This reassignment strategy is explained step by step below:

1. Obtain the minimum observed patrol time received by a quadrant:

$$P_{min} = \min\{P_1, P_2, \ldots, P_J\} \tag{1}$$

   where P_j is the observed police time in quadrant j in our data set.

2. Set a *baseline* (BL) police patrol time for each of the J quadrants of the sample as follows:

$$P_j^{BL,c} = \min\{P_j, P_{min} \times \frac{w_j}{w_{min}} \times \frac{S_j^c}{S_{min}^c}\} \tag{2}$$

   where w_j is the number of street segments covered by quadrant j, w_{min} is the number of street segments covered by the quadrant that reported the minimum patrol time, S_j^c is the share of crimes of type $c \in \{Violent, Property, Overall\}$ that occurred in quadrant j, and S_{min}^c is the share of crimes of type c that occurred in the quadrant that reported the minimum patrol time. That is, we set the *baseline* police patrol time as the minimum between the original police patrol time and the *minimum* police patrol time that is proportional to the number of street segments and the occurrence of crime at each quadrant. This guarantees that the total patrol time assigned is less than or equal to the total patrol time observed. Note that $P_j^{BL,c}$ is indexed by c, which means that we implemented this algorithm three different times for each of the three types of crime.

3. Obtain the residual (RES) time between the observed patrol time and the *baseline* time that will be reassigned, which is given by

$$P^{RES,c} = \sum_{j=1}^{J} \left(P_j - P_j^{BL,c}\right). \tag{3}$$

4. Distribute $P^{RES,c}$ across the first K_x quadrants that report both the highest crime response to police presence (given the *baseline* time assigned) and S_j^c, such that each of the K_x quadrants receive an increase of $x\%$ in the police patrol time that they originally received, while the remaining $J - K_x$ quadrants receive $P_j^{BL,c}$. That is,

$$P_j^{RED,c} = \begin{cases} (1 + x\%)P_j & \text{if} \quad j \in \{1, 2, \ldots, K_x\} \\ P_j^{BL,c} & \text{if} \quad j \in \{K_x + 1, K_x + 2, \ldots, J\} \end{cases} \tag{4}$$

   where $P_j^{RED,c}$ denotes the redistributed police patrol time. Note that K_x is indexed by x. It means that the number of quadrants that receive a $x\%$ increase, K_x, depends on that increase, since $P^{RES,c}$, the available time to be redistributed, is fixed. The greater the percentage increase x, the less quadrants are benefited by the redistribution. Therefore, a trade-off exists in this algorithm between the intensive and extensive margins of police presence across quadrants.

As was noted above, this algorithm was implemented 30 times, one for each $x \in \{10, 20, 30, 40, 50, 60, 70, 80, 90, 100\}$, for each of the three types of crimes that we consider in our work–violent, property, and total crimes. For each crime, we select the $x\%$ increase which results in the lowest level of crimes, and we compare it to the original scenario.
