# Supplementary material for: A location discrete choice model of crime: Police elasticity and optimal deployment
Source: PLoS One. 2024 Mar 12;19(3):e0294020. doi: 10.1371/journal.pone.0294020 (PMC10931527; doi:10.1371/journal.pone.0294020)
Supplement: S1 Fig — α estimated 1,018 times excluding in each iteration one different quadrant. Given that parameter estimates remain stable, IIA assumption seems to hold. (PDF) [file pone.0294020.s002.pdf]

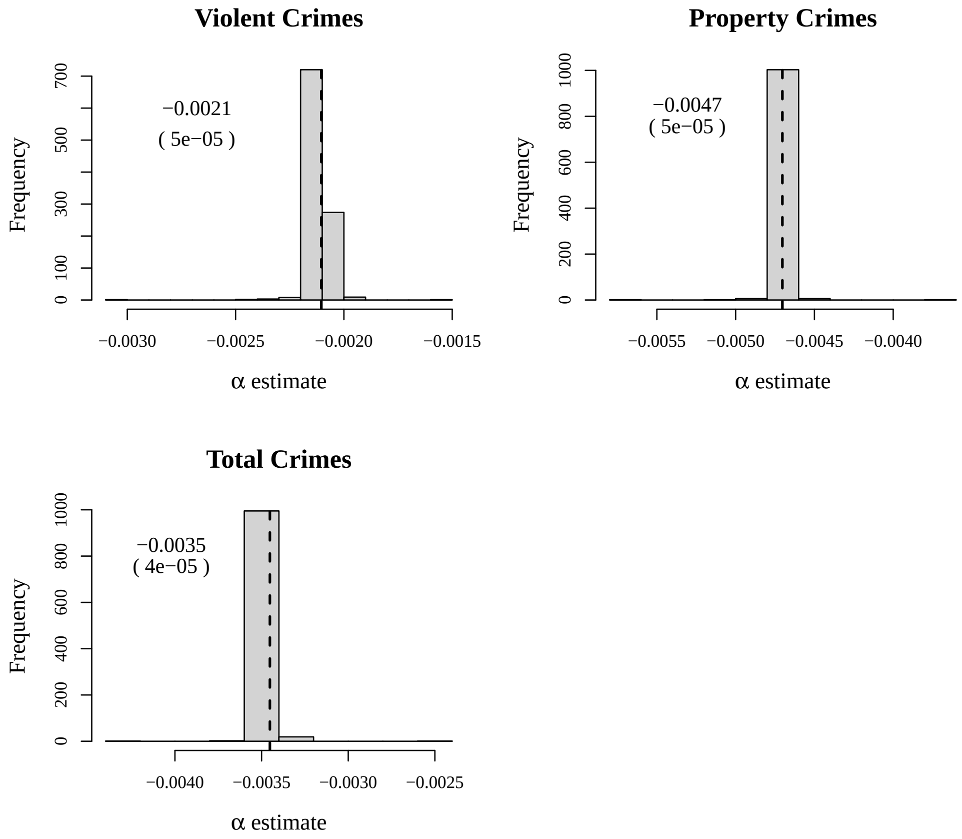

Fig 1. Haussman-McFadden (1984) [27] specification test of the independence of irrelevant alternatives (IIA) assumption. α estimated 1,018 times excluding in each iteration one different quadrant. Given that parameter estimates remain stable, IIA assumption seems to hold. Mean (SD) displayed for each graph. Source: Own elaboration.
