# Supplementary material for: A location discrete choice model of crime: Police elasticity and optimal deployment
Source: PLoS One. 2024 Mar 12;19(3):e0294020. doi: 10.1371/journal.pone.0294020 (PMC10931527; doi:10.1371/journal.pone.0294020)
Supplement: S1 Table — As a robustness check of our results, note that given the simultaneity that exists between crime and police presence, OLS estimates are downward biased. Therefore, if we estimate by OLS we would obtain lower bounds (in absolute value) of the real average impact of police presence on crime. As can be seen form the table, results are indeed downward biased. (PDF) [file pone.0294020.s003.pdf]

Table 1. OLS α estimates of the discrete spatial location choice model. As a robustness check of our results, note that given the simultaneity that exists between crime and police presence, OLS estimates are downward biased. Therefore, if we estimate by OLS we would obtain lower bounds (in absolute value) of the real average impact of police presence on crime. As can be seen form the table, results are indeed downward biased.

	Violent crimes				Property crimes				Total crimes			
	(1)	(2)	(3)	(4)	(5)	(6)	(7)	(8)	(9)	(10)	(11)	(12)
α	-0.000	-0.001**	-0.002**	-0.002**	-0.002**	-0.003***	-0.004***	-0.005***	-0.001*	-0.003***	-0.004***	-0.004***
	(0.001)	(0.001)	(0.001)	(0.001)	(0.001)	(0.001)	(0.001)	(0.001)	(0.001)	(0.001)	(0.001)	(0.001)
Observations	1,050	1,050	1,050	1,050	1,050	1,050	1,050	1,050	1,050	1,050	1,050	1,050
R-squared	0.023	0.316	0.317	0.390	0.019	0.354	0.356	0.424	0.010	0.207	0.211	0.298
Controls	No	Yes	Yes	Yes	No	Yes	Yes	Yes	No	Yes	Yes	Yes
Previos patrol time	No	No	Yes	Yes	No	No	Yes	Yes	No	No	Yes	Yes
Locality FE	No	No	No	Yes	No	No	No	Yes	No	No	No	Yes

Notes: *** p<0.01, ** p<0.05, * p<0.1. Cluster robust standard errors at the level of locality in parentheses.
