# Supplementary material for: A location discrete choice model of crime: Police elasticity and optimal deployment
Source: PLoS One. 2024 Mar 12;19(3):e0294020. doi: 10.1371/journal.pone.0294020 (PMC10931527; doi:10.1371/journal.pone.0294020)
Supplement: Attachment — Submitted filename: Review.docx [file pone.0294020.s004.docx]

**Review**: A Location Discrete Choice Model of Crime: Police Elasticity and Optimal Deployment.

The paper develops a discrete choice model of crime location with aggregate data in the city of Bogota (Colombia). Elasticities are estimated for each of the spatial locations and different police patrolling strategies are evaluated. The paper is interesting and provides a novel analysis for a region where there is not much reliable empirical data for this type of analysis.

That said, I think the paper can be improved in many ways. I am not going to get into the methodological heart of the paper but rather its presentation and conclusions.

1. Although the introduction is quite extensive, I believe that the paper would benefit from the incorporation of a more extensive literature review that would serve to bring the paper into the discussion of these types of models and their relationship to police deployment.
2. The literature review should also incorporate a section on Latin America. In the region there are already papers that analyze crime and police deployment from different methodological perspectives, including at the spatial level (geography of crime). It is necessary to insert this paper in that tradition.
3. Thirdly, it would be interesting to incorporate a discussion where the results are analyzed and then the conclusions where these results are connected with other literature, a future line of research is proposed to expand this analysis.
4. However, beyond these particular issues, the paper seems to move between two literatures that are not integrated in the text and neither at the level of analysis of results and conclusions: location of police resources and location of crime or concentration of crime. As it is, it is difficult to distinguish whether it is a contribution to the study of crime concentration patterns in large Latin American cities or whether it is intended as a contribution to the discussion on police distribution. In short: its results, which are valuable, are lost in part because they are not oriented to a particular debate.
5. There are no real conclusions in the paper either. A complete section should be included where the results are used to draw public policy conclusions on the level of concentration and distribution of crime or police deployment.

For the rest, I am grateful for the opportunity to have read this contribution, which can be a contribution to the discussion of crime in Latin America.
